# Uterine Torsion Dystocia Complicated by Perosomus Elumbis in an Angus Calf Associated with a Consanguineous Mating

**DOI:** 10.1155/2020/6543037

**Published:** 2020-02-10

**Authors:** Alyssa B. Helms, Riley E. Thompson, Sam Lawton, Jessica L. Petersen, Allison Watson, Mee-Ja Sula, David Steffen, Brian K. Whitlock

**Affiliations:** ^1^Department of Large Animal Clinical Sciences, College of Veterinary Medicine, University of Tennessee, Knoxville, TN 37996, USA; ^2^University of Nebraska-Lincoln, Lincoln, NE 68583, USA; ^3^Department of Biomedical and Diagnostic Sciences, College of Veterinary Medicine, University of Tennessee, Knoxville, TN 37996, USA

## Abstract

A six-year-old multiparous Angus cow was presented for dystocia. Vaginal and rectal examinations revealed an approximately 360° counterclockwise uterine torsion. The torsion was corrected by rolling the cow counterclockwise (three episodes) with the aid of a plank coupled with manual detorsion via the vagina. The placement of obstetric chains followed by manual traction ultimately delivered a stillborn male calf with evidence of vertebral aplasia, arthrogryposis, and abdominal organ herniation. Patient history and subsequent parentage verification revealed that the calf was the result of a consanguineous (mother to son) mating. Tissue samples from the affected calf and blood samples from the dam, sire, and ten half siblings were collected for genetic testing and parentage verification. Necropsy, radiographic, and computed tomography examinations all supported a diagnosis of perosomus elumbis. Perosomus elumbis is a congenital abnormality of unknown origin(s), and this is the first report of a case associated with a consanguineous mating.

## 1. Introduction

Perosomus elumbis (PE) is a congenital abnormality of unknown origin(s) that is characterized by aplasia of the lumbar vertebrae and spinal cord. Perosomus elumbis has been reported in several domestic species, including cattle. While this condition has been reported in Holsteins and Herefords, it has not yet been reported in Angus cattle. One case of PE was associated with a Holstein calf infected with bovine viral diarrhea virus (BVDV). This is the first report of PE associated with a consanguineous mating.

## 2. Case Presentation

A six-year-old female multiparous Angus cow, weighing approximately 800 kg with a history of five parities, presented at the farm of origin on emergency for dystocia. The cow had begun stage two labor earlier that morning but failed to progress at which time the owner contacted the University of Tennessee Veterinary Medical Center (UTVMC). Upon arrival at the farm, the cow was restrained in a chute. She was bright, alert, and responsive with a normal and relaxed posture. To facilitate vaginal and rectal examination, the attending veterinarian administered a caudal epidural anesthetic of 5 mL of lidocaine HCl (20 mg/mL, dosed at 0.13 mg/kg). The labia and perineum were cleaned, and an obstetric sleeve and lube were used for manual vaginal examination. On vaginal palpation, the cranial vagina was more narrow than expected and the cervix was dilated approximately 6 to 8 cm. Gentle digital pressure allowed for the passage of a hand through the cervix where two forelimbs and a tightly constricted uterus were palpated. “Rifling” of the vagina was not appreciated. Subsequent transrectal palpation of the uterus revealed that the right broad ligament was felt as a taught band and traced dorsally from the right side, ventrally to the left. Based upon the manual vaginal and transrectal palpation examinations (especially positioning of the broad ligaments, uterus, and fetus), the cow was diagnosed with an approximately 360° left (counterclockwise from the rear of the animal) uterine torsion.

The cow was sedated with intravenous administration of 40 mg of xylazine HCl (20 mg/mL, dosed at 0.05 mg/kg) and 20 mg of acepromazine maleate (10 mg/mL, dosed at 0.025 mg/kg), casted using the double half hitch method, and placed in left lateral recumbency. The uterine torsion was partially corrected by rolling the cow counterclockwise with the aid of a plank (three episodes) and ultimately completely corrected (final 90°) by manual detorsion via the vagina. A vaginal examination of the cow revealed a partially dilated cervix (8 to 10 cm), a moderately contracted uterus, and a fetus in cranial longitudinal presentation and dorsosacral position, with the head, neck, and forelimbs extended. A 10 mg injection of epinephrine HCl (1 mg/mL, dosed at 0.013 mg/kg) was administered intravenously to the cow as a tocolytic to better allow for manipulation of the fetus and placement of obstetric chains and snare to the forelimbs and head, respectively. Manual traction, for a duration of approximately thirty minutes by two individuals, completely dilated the cervix and ultimately delivered a stillborn male calf presumptively diagnosed with PE. A vaginal examination revealed no additional fetuses and minimal partial thickness uterine and vaginal tears. A single 5.3 g injection of ceftiofur crystalline free acid (200 mg/mL, dosed at 6.6 mg/kg) was administered subcutaneously in the base of the left ear, and a single dose of 1.5 g of flunixin meglumine (50 mg/mL, dosed at 1.9 mg/kg) was administered intravenously to the cow to provide prophylactic broad-spectrum antimicrobial and anti-inflammatory/analgesic treatment, respectively.

The cow recovered uneventfully from the dystocia, and thorough history revealed that the calf was the result of an unintentional, consanguineous, mother to son mating. Tissue samples from the affected fetus and blood samples from the dam, sire, and ten half siblings were collected. Relational status was confirmed via parentage verification utilizing the commercially available SeekSire™ parentage assay (Neogen Genomics) which compares over 200 single nucleotide polymorphism genotypes to confirm identity by descent. No history of teratogen exposure was reported, and testing of the calf by antigen capture enzyme-linked immunosorbent assay for BVDV was negative. The herd was maintained as a closed herd, receiving annual routine immunizations as are recommended for the region, with breeding practices including fixed timed artificial insemination followed by exposure to bulls for natural breeding.

**Figure 1:**
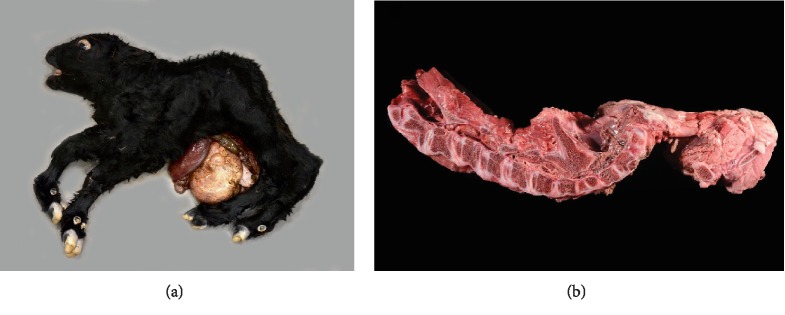
Gross necropsy photos of the stillborn male Angus calf with appreciable congenital deformities: short and rounded skull, maxillary brachygnathism, arthrogryposis, vertebral aplasia, and abdominal organ herniation (a). A longitudinal section of the spine in right lateral orientation (b) highlights agenesis of the lumbosacral and caudal vertebra and spinal cord agenesis abnormalities as are characteristic of perosomus elumbis.

**Figure 2:**
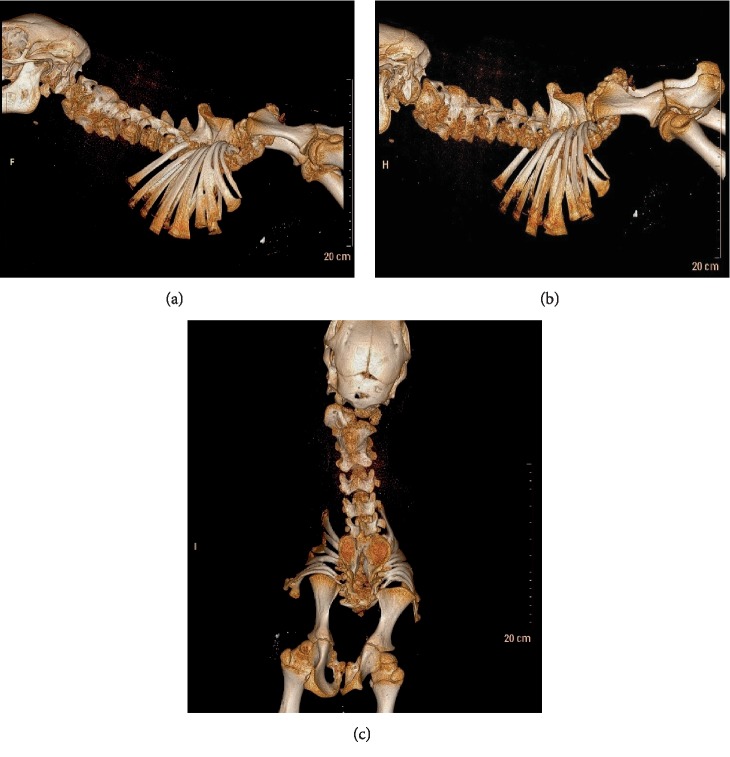
Right lateral (a), left lateral (b), and dorsoventral (c) computed tomography images of the calf revealing congenital partial lumbar and complete sacral and coccygeal vertebral aplasia consistent with perosomus elumbis.

The stillborn calf was submitted for necropsy to the UTVMC. Gross necropsy observations revealed a 19.2 kg male calf (Figure 1). The calf had a skull that appeared short and rounded with maxillary brachygnathism and bilateral exophthalmos. Bilaterally symmetric arthrogryposis and partial lumbar and complete sacral and coccygeal vertebral aplasia (consistent with PE) were observed. The present lumbar vertebrae were fused with no intervertebral disc spaces. The spinous processes of the thoracic vertebrae were fused for a total of two spinous processes with eight rib pairs causing diverging, rather than parallel, ribs. An 18 cm long abdominal midline defect was observed with abdominal organ herniation and atresia ani; otherwise, all abdominal organs were present. Radiographic and computed tomography examinations were consistent with and supported the diagnosis of PE (Figure 2).

## 3. Discussion

Perosomus elumbis is a congenital bovine fetal anomaly characterized by a group of defects including agenesis of the lumbosacral and caudal vertebra, spinal cord agenesis, arthrogryposed or ankylosed hypoplastic caudal limbs, and malformations of the abdominal viscera and urogenital and anorectal systems [1]. Well known to occur in Holstein and Herford cattle, PE has also been reported in sheep, horses, pigs, and a dog [2–7]. To date, the cause of PE is unknown, as the literature on this topic has primarily consisted of case reports. Prior causal speculations have included teratogens such as Veratrum californicum, fetal infection with BVDV, and a possible genetic etiology [2, 3, 8]. The genetic etiology has been highly considered, as similar sires have been reported within the pedigrees of affected Holstein calves [2]. One hypothesis further suspects a mutation within the homeobox gene family which is expressed in the developing nervous and axial skeletal systems; however, genetic testing for such a chromosomal abnormality for PE has yet to be accomplished [9]. This is the first published report of PE in Angus cattle as a result of a consanguineous, mother to son mating. Given the close inbreeding, it is possible that this case of PE may reveal more information regarding the hypothesized genetic etiology. Blood and tissue samples from the affected calf, dam, sire, and multiple half siblings have been collected, and subsequent genetic analyses are pending.
